# Secret of Success in Medical Practice

**Published:** 1847-04

**Authors:** 


*Secret of Success in Medical Practice.*—Skill and art alone are not sufficient. He (a medical practitioner) must be particularly mindful of his conduct. It is this which recommends him to the public, and creates confidence; for as the generality of people are incompetent to pronounce on his science, it is natural for them to take the measure of his ability from the measure of his conduct. By force of conduct alone, a physician, of very moderate talents, may become the favourite of the public; and without it, the most skilful professional man remain unnoticed and unappreciated.—*Lond. Med. Gaz. from Hufeland.*

